# Motor and non-motor fluctuations in Parkinson’s disease: the knowns and unknowns of current therapeutic approaches

**DOI:** 10.1007/s00702-025-02990-4

**Published:** 2025-07-28

**Authors:** Martin Regensburger, Ilona Csoti, Wolfgang H. Jost, Zacharias Kohl, Stefan Lorenzl, David J. Pedrosa, Paul Lingor

**Affiliations:** 1 Department of Molecular Neurology, Friedrich-Alexander-Universität Erlangen-Nürnberg, Erlangen, Germany; 2 Fachklinik für Parkinson, Gertrudis Klinik Biskirchen, Leun-Biskirchen, Germany; 3 Parkinson-Klinik Ortenau, Wolfach, Germany; 4 Department of Neurology, University of Regensburg, Regensburg, Germany; 5 Institute of Palliative Care, Paracelsus Medical University, Salzburg, Austria; 6 Department of Neurology and Palliative Care, Krankenhaus Agatharied, Hausham, Germany; 7 Department of Palliative Care, Ludwig-Maximilians University Munich, Munich, Germany; 8 Department of Neurology, Philipps-University Marburg, Marburg, Germany; 9 Department of Neurology, Klinikum rechts der Isar, School of Medicine and Health, Technical University Munich, Munich, Germany

## Abstract

Neurodegeneration in Parkinson’s disease is chronically progressive, and no disease-modifying therapies have been approved so far. Fluctuations emerge in eventually all people with Parkinson’s disease, and may lead to a high burden of motor and non-motor disability and significantly impair participation if they are inadequately treated. In recent years, the range of therapeutic options has expanded considerably. While different types of oral dopaminergic substances are initially applied to control fluctuations, additional routes of administration now encompass sublingual, inhalative, subcutaneous and transdermal applications. Different choices exist for on-demand and continuous pump therapies, as well as for deep brain stimulation. In this narrative review, we summarize the state of the art in the identification and treatment of motor and non-motor fluctuations in Parkinson’s disease. Moreover, we discuss practical aspects of managing fluctuations, address yet unresolved questions and we offer insights into upcoming clinical developments.

## Introduction

Parkinson’s disease (PD) is the second most common neurodegenerative disorder. With an estimated prevalence of more than 6 million people affected worldwide (GBD et al. 2017), and an overall increased longevity, it is expected to cause a massive increase in cases (Dorsey et al. 2018). Moreover, additional environmental factors lead to an increased incidence (Ben-Shlomo et al. 2024). In the early stages of the disease, very mild symptoms may not require medication or can be effectively controlled with levodopa, dopamine agonists or the MAO-B-inhibitor rasagiline. However, as the disease progresses, fluctuations complicate the disease course further (Stocchi et al. 2024). While a disease modifying therapy for PD is still lacking, there is a plethora of treatments available for fluctuations in PD. Hence, screening for or dealing with fluctuations in PD constitutes a frequent reason for consultation in neurological practice. In the present review, we outline current concepts of diagnosing and treating fluctuations in PD, and we address open questions and how they could be answered in the future.

### Definition of fluctuations in Parkinson’s disease

Fluctuations refer to changes in disease-related symptoms during day or night (Foltynie et al. 2024). Motor fluctuations are most evident to the examiner, and have been classified into hypo- and hyperkinetic states. *Box 1* summarizes motor as well as non-motor fluctuations that are frequently encountered.

**Table:** Box 1 Classification of fluctuations in Parkinson’s disease, adapted from Chou et al. 2018 (Chou et al. 2018)

Motor fluctuations	- Early-morning akinesia / bradykinesia - Wearing off / end-of-dose deterioration - On-off phenomenon / sudden off / random off - Delayed “on” - Dose failure / no “on” - Weak response at the end of the day - On-dyskinesia / peak-dose dyskinesia - Biphasic dyskinesia
Non-motor fluctuations	- Pain and sensory - Orthostatic hypotension - Fatigue - Sleep disturbance - Cognitive symptoms - Affective fluctuations, anxiety

### Mechanisms of fluctuations in Parkinson’s disease

Different mechanisms that are partly overlapping have been proposed to explain the circadian variability of response to dopaminergic medication during the progression of PD, and levodopa treatment plays a central role (Riederer et al. 2025). According to the most favored *storage hypothesis*, the progressive loss of dopaminergic presynaptic terminals stemming from the nigrostriatal tract leads to a decreasing capacity of dopamine storage in the striatum (Fabbrini et al. 1988; Chase et al. 1989). Pharmacologically active levodopa is taken up by remaining dopaminergic presynapses where it is decarboxylated intracellularly and stored in presynaptic vesicles to enable tonic and phasic release patterns of dopamine (Mosharov et al. 2015). This concept overlaps with the clinically observed short duration response vs. long duration response upon acute vs. chronic levodopa administration (Quattrone et al. 1995). At the clinical motor onset of PD, however, more than 60% of dopaminergic terminals in the substantia nigra have already been lost, meaning that release of levodopa-derived dopamine from nigral neurons is profoundly different from the physiological dopaminergic stimulation (Burke and O’Malley 2013). With ongoing neurodegeneration, the buffering function of dopaminergic neurons diminishes even more and serotonergic neurons that are initially spared in the disease process take up levodopa as a mal-adaptice plasticity contributing to dyskinesia (Rylander et al. 2010; Sulzer and Surmeier 2013). As a second theme, a *pulsatile dopaminergic stimulation* has been linked to motor fluctuations (Chase et al. 1989; Stocchi 2009). Consistently, a PET study revealed that a single levodopa dose led to substantially higher synaptic dopamine levels in patients with motor fluctuations compared to those without motor fluctuations (Fuente-Fernández et al. 2001). A pulsatile stimulation may also contribute to a certain degree of levodopa-induced toxicity linking high levels of levodopa to earlier emergence of motor fluctuations. Excessive oxidation as a result of non-physiological dopamine concentrations leads to mitochondrial and lysosomal impairment which may initiate a vicious cycle in PD (Burbulla et al. 2017; Prasuhn et al. 2024). Finally, peripheral *alterations in levodopa pharmacokinetics* explain unsteady CNS concentrations of levodopa. This includes transport and uptake irregularities as a consequence of progressive autonomous involvement in PD such as different types of swallowing deficits in PD as well as reduced gastric emptying leading to delayed “on” or dose failures (LeWitt 2015; Labeit et al. 2022). Different amino acids compete with levodopa during its uptake in the intestine and when crossing the blood brain barrier. This is why protein-rich nutrition constitutes another reason for a reduced or delayed uptake of levodopa from the gut to the brain (Nutt et al. 1984; Leenders et al. 1986). Alterations of the gut microbiome in PD are still incompletely understood, but eradication of Helicobacter pylori might provide a more stable uptake of levodopa (Rees et al. 2011; Cheng et al. 2024). Taken together, all of these aspects reinforce the concept of a continuous dopaminergic stimulation in order to delay and to counteract motor fluctuations in PD (Pirtošek et al. 2023). Finally, in later stages of PD, off phases may be resistant even to continuous dopaminergic therapies, arguing for a role of *additional neurotransmitters* including serotonin and noradrenaline (Rota et al. 2022).

Motor fluctuations are frequent. A metaanalysis estimated that motor fluctuations emerge in 40% of patients after 4–6 years of disease duration (Ahlskog and Muenter 2001). In trials with initiation of levodopa, they occurred even earlier, for example in 20% of levodopa treated PD patients after 9 months (Fahn et al. 2004) and in 75% of PD patients after initiation of levodopa in the STRIDE-PD trial (Olanow et al. 2013). In line with the aforementioned mechanistic concepts, disease progression is the most important factor associated with motor fluctuations, but additional risk factors have been identified which are also related to total levodopa dosage (*Box 2*). Hence, current guidelines recommend avoiding early oral (i.e., phasic) administration of excess doses of levodopa (Fox et al. 2018; Pringsheim et al. 2021; Höglinger et al. 2024).

**Table:** Box 2 Factors that have been associated with the development of motor fluctuations in Parkinson’s disease (Olanow et al. 2013; Sun et al. 2020)

- Young age at onset - Higher doses of levodopa - Lower body weight - Increasing disease duration - Female sex - Increased disease severity (UPDRS parts II and III)	

Levodopa is still the main substance in drug treatment of PD. However, its half-life is limited between 50 and 90 min. In combination with a decarboxylase inhibitor, its effectiveness can be increased to about 2 h as of yet. Thus, it must be administered several times per day to maintain a patient’s functionality throughout their daily lives as much as possible. There is a time between the diminishing effect of a previous dose (‘wearing-off’) and the onset of effect of the next dose, during which symptoms can naturally increase. These are often called ‘off-phases’ or ‘off-periods’. In other words, the day of a PD patient with fluctuations is marked by changes in drug effectiveness and therefore in the severity of their symptoms.

According to a Delphi panel consensus of 17 international PD experts, 6 out of 15 indicators of “advanced PD” were related to the presence of motor or non-motor fluctuations (Antonini et al. 2018). Hence, the term “advanced stage” PD may imply a negative connotation when referring to motor fluctuations. Nevertheless, motor fluctuations may be a feature that is present early in the disease course which is why treatment options for “advanced PD” should not be withheld from this population, even more so as data on their long term treatment have increasingly become available.

### Questionnaire based methods to identify and to quantify fluctuations

Patients may experience difficulties in recognizing or articulating changes in their symptomatology or disease state. Furthermore, many relevant phenomena are not readily observable by medical staff during routine examinations, particularly in the early disease stages. Accordingly, the assessment tools discussed below can serve as valuable adjuncts to the diagnostic process and contribute to a more informed treatment planning. Despite the availability of innovative technologies for detecting and quantifying symptom fluctuations, interpretation and clinical contextualization remain tasks best performed by the clinician responsible for long-term patient care (Müller et al. 2024).

As in many other medical areas, standardized questionnaires are the most widely used instruments for capturing symptom fluctuations, owing to their efficiency and ease of use. Standardization enhances objectivity and comparability across individuals. The variety of questionnaires presented below differs mainly in the number of questions they include, which makes them suitable for more specific uses: from a quick screening tool with just 9 symptoms to more in-depth examinations. Some questionnaires need to be completed by physicians while others can be filled out by the patients. However, their use can be limited by recall bias and, in some cases, by restricted access due to licensing requirements.

One widely used tool is the *Movement Disorder Society Unified Parkinson’s Disease Rating Scale* (MDS-UPDRS), a revised and expanded version of the original UPDRS. It assesses various aspects of the disease across four domains, with the fourth part containing questions specifically about drug effects, motor fluctuations and dyskinesia (Goetz et al. 2008). The *Unified Dyskinesia Rating Scale* (UDysRS), in turn, has been specifically developed as a tool to quantify hyper- and dyskinesia (Stacy 2010).

Another frequently used instrument is the *Wearing-Off Questionnaire* (WOQ-32), a patient-completed tool introduced in the 2000s to assess both motor and non-motor symptoms associated with wearing-off phenomena (Stacy et al. 2005). The authors later developed significantly shorter versions for use in routine clinical settings such as the WOQ-19 and the WOQ-9, the latter focusing on the nine most predictive symptoms of wearing-off (Stacy et al. 2006; Stacy and Hauser 2007).

The *Non-Motor Fluctuation Assessment* (NoMoFA) is another patient-reported questionnaire, designed specifically to capture 28 non-motor symptoms related to off periods. In addition, it evaluates the impact of these symptoms on quality of life and their fluctuation in relation to levodopa administration (Kleiner-Fisman et al. 2011).

A different diagnostic tool that can be used in addition to structured questionnaires is a diary that patients keep themselves. They can be particularly beneficial when the causes of fluctuations or other medical problems are not immediately attributable or standard clinical interventions are insufficient. By documenting symptoms and motor events along with the situational contexts and potential triggers, patients provide clinicians with rich, individualized data that can inform diagnostic and therapeutic counselling. Moreover, the act of recording can also foster a heightened awareness for potential issues in daily routines on the side of the patients and their families, which in turn can increase self-efficacy in finding solutions and in the treatment process. Thus, diaries have become a popular diagnostic and therapeutic tool in many areas of the healthcare system. They don’t have to be traditional diaries either. Simplified formats such as tables with brief annotations as shown below, may suffice for many patients.

**Table:** 

Day	Time	Last dose	Location	Description of event
*Monday*	*9.30 AM*	*8 AM (50mg)*	*Supermarket*	*Small steps*,* risk of falling*
…	…	…	…	…

Specifically for visualizing drug effects and symptom fluctuations over the course of the day the Hauser diary was used in studies for many years, where patients or their relatives documented on- and off-phases and dyskinesias (Hauser et al. 2000). The disadvantage of this diary was its subjectivity and its dependence on the cognitive ability of the study participants to perceive symptoms accurately and objectively over time. Therefore, it was eventually replaced by electronic diaries which can, for example, include medication alarms as well (Rovini et al. 2018; Terroba-Chambi et al. 2018).

Finally, the Well-Being Map™ is an 8-domain patient questionnaire to identify motor and non-motor symptoms in PD, offering a structured tool for evaluation (Skogar and Nilsson 2018).

### Digital quantification of fluctuations

Fluctuations represent an ideal target for digital tracking, at least for motor fluctuations, but potentially also for non-motor changes. There is a growing shift toward sensor-based, automated, and objective monitoring of PD symptoms using digital devices. Several systems have been developed for this purpose, with some already approved in the United States and the European Union (Moreau et al. 2023). However, the current evidence regarding their clinical validation, user satisfaction, usability, and overall added clinical value remains limited or inconsistent (Reichmann et al. 2023). They may be used as outcome measures in clinical trials and as feedback tools for patients and caregivers to monitor motor function. While a detailed description of all available systems goes beyond the scope of this review, the following section provides a representative selection and classification of digital monitoring approaches (Ossig et al. 2016a). These can broadly be categorized based on the sensing methodology.

*Non-wearable systems*, such as home-installed sensors, based on millimeter-wave radar technology, offer a highly unobtrusive option for long-term monitoring. These systems emit and sense radar signals reflected by moving objects and bodies and allow continuous observation over weeks or months (Krauss et al. 2024). In a pilot study involving 32 PD patients, a single radar device installed at home was able to detect gait speed and estimate the degree of motor fluctuations (Liu et al. 2022). Nevertheless, follow-up trials and further assessments of privacy concerns and real-world applicability are still needed.

Classical *wearable systems* predominantly utilize inertial measurement units to capture motion patterns. Advanced algorithms and rigorous clinical validation are required to infer motor states from these agnostic sensors. Initial clinical development of these monitoring tools primarily focused on basic motor features such as gait speed and step counts (Polhemus et al. 2021). Devices vary considerably in terms of sensor type, number of sensors worn, and duration of recording. Foot-worn sensors such as the Stepmetric system (Portabiles-HCT) have proven effective in assessing gait impairment and, more broadly, motor state (Schlachetzki et al. 2017; Jakob et al. 2021). The APDM Mobility Lab Opal system can detect medication response but is primarily designed for in-clinic assessments (Curtze et al. 2015). Recurring, *task-based motor assessments* offer an additional strategy for longitudinal at-home monitoring. The Koneksa system integrates a smartphone app and smartwatch with motor tasks, providing snapshot evaluations of diurnal fluctuations. While this approach may be especially relevant in trials of disease-modifying therapies during early PD stages, it offers limited continuous monitoring (Lavine et al. 2024). The DynaPort/McRoberts system, while not specifically developed for PD, consists of a single sensor worn at the lumbar spine or wrist and has been used successfully in this context (Kirk et al. 2024). Several commercial insole-based systems such as FeetMe are also emerging for PD gait monitoring (Parati et al. 2022). The Adamant Health sensing system is an example of skin-contact electrodes for *additional surface EMG* recording which might enable a more detailed characterization of tremor, rigidity, and bradykinesia (Rissanen et al. 2021). Such systems may be particularly helpful for individuals who struggle to self-identify or report motor fluctuations reliably (e.g., in distinguishing tremor from dyskinesia). Integration of these systems with patients’ smartphones and smartwatches has shown promise for enhancing usability and reducing the sensor burden, particularly during early, drug-naïve stages of PD (Adams et al. 2024).

Some systems have specifically addressed monitoring of motor fluctuations. The PDMonitor system, which includes five sensors worn throughout the day, demonstrated good correlation between algorithm-derived measures and patient- or clinician-reported OFF times and dyskinesia (Antonini et al. 2023b). The STAT-ON device, a single waist-worn measurement unit, showed reliable detection of motor fluctuations and responsiveness to treatment changes over multi-day use (Caballol et al. 2023). In a 12-month controlled study of 40 PD patients, use of the finger-worn Kinesia system in combination with structured motor tasks led to an increased rate of referrals for advanced PD therapies (Heldman et al. 2016). Similarly, symptom monitoring with the wrist-worn Parkinson’s KinetiGraph and physician feedback resulted in improved symptom control (Woodrow et al. 2020).

In addition to external sensors, invasive systems now allow direct recording of local field potentials (LFPs) via certain deep brain stimulation (DBS) implants. One such system is now FDA-approved for triggering adaptive stimulation (Shukla et al. 2025). In particular, subthalamic beta-band power (13–30 Hz) correlates with bradykinesia and rigidity and responds to both dopaminergic medication and stimulation, making it a key biomarker for closed-loop DBS (Busch et al. 2023; Oehrn et al. 2024). As such, invasive quantification offers a robust physiological readout of motor state in patients that have undergone DBS while the exact titration of medication vs. stimulation adaptation remains complex.

More recently, efforts have shifted toward implementing fluctuation-relevant algorithms and integrating these technologies into clinical care pathways. For example, the ParkinsonGO platform is partially reimbursed by health insurers in Germany, connecting home-based monitoring with PD nurses and the treating neurologist. However, its precise effects on the overall care process and healthcare costs are still being analyzed in the European PDnetGo consortium (Study registry: DRKS00034945).

### Diagnostic workup and basic therapeutic adjustments

A general objective in the pharmacological management of PD is to ‘smooth out’ motor fluctuations as much as possible by optimizing the intake schedule and dosage. This involves balancing the reduction of off-phases with the prevention of unnecessary dosing and the associated side effects, especially in later stages of the disease. Achieving this requires a broad understanding of the various off-symptoms and other factors that can contribute to fluctuations (like drug interactions or protein intake from food for example), as well as detailed insight into each patient’s daily routines and fluctuations in order to make personalized treatment plans.

When fluctuations are suspected in PD (*Box 1*), adherence to the current dosing plan should first be inquired, as well as nutrition (time interval of levodopa to meals, avoidance of excess protein) and indicators of gastrointestinal function (dysphagia, presence of regurgitation, constipation). Swallowing dysfunction is a common concern, affecting up to 70% of PD patients. In such cases, diagnostic confirmation may be obtained via flexible endoscopic evaluation or videofluoroscopic assessment of tablet and capsule ingestion (Labeit et al. 2022). As swallowing impairments may reflect an off-state manifestation, they may warrant adjustment of dopaminergic therapy. Regardless of etiology, the initiation of swallowing therapy is strongly recommended. In cases of delayed gastrointestinal transit, therapeutic options include dietary adjustments (e.g., small, frequent meals), the use of peripheral dopamine antagonists such as domperidone, motilin receptor agonists (e.g., erythromycin, azithromycin), serotonin receptor agonists (e.g., mosapride, prucalopride), cholinergic agents (e.g., bethanechol, pyridostigmine), and adjunctive treatments such as probiotics and laxatives (Leta et al. 2023).

### End-of-dose phenomena

Distinct from the early morning off phase, end-of-dose is characterized by the predictable recurrence of motor and non-motor symptoms associated with a decreasing effect of the last intake of dopaminergic medication. Patients are often actively aware of these “wearing off” episodes during the day. However, defining an off state remains highly individual since its timing, symptomatology, and perceived severity may vary widely between patients (Antonini et al. 2011). Importantly, classical wearing-off questionnaires such as the WOQ-32 do not provide a clear separation between end-of-dose phenomena from random on-off, delayed-on and no-on episodes, and also clinically, off phases rather represent a spectrum than clearly distinct entities (Chou et al. 2018).

### Random-off episodes

Random-off or unpredictable off episodes occur without a consistent temporal relation to the intake of dopaminergic medication. Due to their unanticipated nature, they are particularly distressing for patients. The underlying pathophysiology has been linked to factors that are also relevant for wearing-off, i.e. erratic gastric emptying, delayed intestinal absorption, unstable blood-brain barrier transport, and dopamine receptor changes (Chou et al. 2018). Clinically, they may manifest as sudden freezing, sudden deterioration of rigidity, bradykinesia or severe non-motor symptoms. Their therapy overlaps with the therapy of motor fluctuations in general and on-demand therapeutic options can be useful.

### Management of early morning-off

Early morning-off episodes often represent the initial clinical manifestation of emerging motor fluctuations in PD. These episodes are most commonly associated with low plasma levodopa concentrations upon waking and typically resolve following the first morning dose of medication (Chou et al. 2018). Patients may describe morning-off periods as difficulty in performing routine activities of daily living, such as rising from bed, using the bathroom, dressing, and experiencing associated musculoskeletal pain (Onozawa et al. 2016).

The presence of significant early morning-off symptoms should prompt a reassessment of treatment strategy. A range of therapeutic options has demonstrated efficacy in this context, and evidence is available for several pharmacological interventions (Kataoka and Sugie 2024). Prompt administration of oral levodopa immediately upon waking is recommended, with dispersible formulations showing superior benefit compared to standard preparations (Steiger et al. 1992). In cases where patients experience nocturnal hypokinesia or sleep disturbances, the addition of extended-release levodopa at bedtime may further improve morning motor function.

Clinical studies have specifically demonstrated positive effects on early morning-off symptoms for several prolonged-release dopamine agonists, including pramipexole, ropinirole, and rotigotine (Schapira et al. 2011; Yun et al. 2013; Vallderiola et al. 2015; Pierantozzi et al. 2016). Among the classical non-ergoline dopamine agonists, rotigotine offers continuous drug absorption through its transdermal delivery system. This administration route may be beneficial in specific clinical scenarios, such as in patients with swallowing difficulties (including perioperative or nil-by-mouth conditions) or those with nocturnal symptoms (Raeder et al. 2021). Compared to the extended-release formulations of pramipexole and ropinirole, rotigotine’s shorter half-life allows for a relatively quicker titration (Jost et al., 2024). It may also be considered overnight-only, as part of a strategy to manage early morning OFF episodes, particularly in combination with subcutaneous apomorphine or intrajejunal levodopa infusions that are paused overnight (Lau et al. 2022). Additionally, the catechol-O-methyltransferase (COMT) inhibitor opicapone (Bologna et al. 2024; Hauser et al. 2024) and monoamine oxidase-B (MAO-B) inhibitors safinamide and rasagiline (Stocchi et al. 2015; Takeda et al. 2022) have shown efficacy for reduction of early morning off episodes.

In cases requiring rapid symptom relief or when gastrointestinal absorption is compromised, non-oral, on-demand therapies are a valid next option. Regarding reduction of early-morning off phases, specific evidence exists for subcutaneous apomorphin pen injections (Gancher and Nutt 1987; Isaacson et al. 2017), sublingual apomorphine film (Hauser et al. 2016; Isaacson et al. 2022) and inhaled levodopa (Hauser et al. 2019; Jost et al. 2023). Moreover, all device-aided therapies have been associated with a reduction in off time, with specific improvements in early morning motor function documented in studies on DBS (Cubo et al. 2001; Lyons and Pahwa 2006) and in the pivotal trial of foslevodopa/foscarbidopa infusion therapy (Soileau et al. 2022). In summary, early morning-off is a frequent sign of evolving motor fluctuations and should be actively inquired and treated in daily PD practice.

### Nocturnal fluctuations

Sleep disorders in PD are complex and closely linked to both motor and additional non-motor symptoms (Dodet et al. 2024). Sleep disorders include difficulties initiating sleep, nocturnal cramping, painful dystonia and impaired mobility in bed, such as turning over or rising to urinate. These symptoms may be exacerbated by coexisting urinary dysfunction, confusion, or hallucinations (Poewe 2008). As a consequence, daytime impairments such as excessive daytime sleepiness and sudden sleep attacks may be worsened.

In a significant portion of patients, these disturbances are associated with nocturnal motor fluctuations. In the PRIAMO study, more than 78% of PD patients with motor fluctuations reported sleep disturbances (Barone et al. 2009). Anatomically, fragmented sleep was linked to altered activity in the medial prefrontal cortex, where increased neural activity during sleep correlated with both sleep disruption and worsening of motor symptoms (Wang et al. 2025).

Sleep disturbances often occur in patients with day-time fluctuations or dyskinesia. The recent OASIS trial demonstrated the efficacy of opicapone in improving sleep disturbances in PD (Ferreira et al. 2024), consistent with findings for other COMT inhibitors (Ebersbach et al. 2010; Park et al. 2020). Similarly, safinamide has also been shown to improve both nocturnal fluctuations and sleep quality (Bovenzi et al. 2024).

### Fluctuations of non-motor symptoms in PD

Besides the characteristics of motor symptoms and their various aspects of fluctuation in advanced stages of PD, a large variety of non-motor symptoms (NMS) appear to be highly prevalent, impairing patients’ quality of life. The significance of NMS may outway the burden of motor symptoms, particularly in later stages of the disease (Hely et al. 2005; Chaudhuri et al. 2015). Some of the first fluctuations of NMS described were alterations of mood in patients in motor OFF states (Marsden and Parkes 1976). Nowadays, it is generally accepted that non-motor fluctuations (NMF) do not represent just reactions to motor OFF states, nonetheless co-occurrence is frequent. Cross-sectional data from 464 PD patients suggested that NMF mainly occur in patients suffering from motor fluctuations as well, only a small proportion (7%) showed NMF only (Seki et al. 2013). More recent data imply that in most patients NMF occur in parallel or rather after the first emergence of motor fluctuations: Kim et al. followed up a cohort of 212 PD patients without fluctuations. About half of the participants developed motor fluctuations within 3 years, with 27,4% simultaneous occurrence of NMF and 21,2% later emergence of NMF. Only 1,4% exhibited NMF prior to motor fluctuations (Kim et al. 2018). Fatigue and sleepiness, anxiety and sadness as well as cognitive slowing were the earliest NMF mostly reported (Kim et al. 2018). Interestingly, close temporal connections between motor fluctuations and NMF were only present for mood and cognitive symptoms, with low rates of simultaneous switches between ON and OFF states for motor symptoms and NMS (Ossig et al. 2016b).

NMS and NMF both severely affect quality of life (Storch et al. 2013; Chaudhuri et al. 2015; Martínez-Fernández et al. 2016), but in contrast to the knowledge from motor fluctuations, the additional burden of NMF beyond that induced by NMS is rather limited. At least, the presence of motor fluctuations seems to lead to an increased burden of NMS in PD patients (Santos-García et al. 2022). As NMS comprise a wide range of different symptoms, it appears useful to cluster symptoms in categories, e.g. neuropsychatric, sensory and autonomous NMS (Seki et al. 2013; Classen et al. 2017). Moreover, it is crucial to use standardized measures to assess the occurrence and the severity of NMS, and subsequently of NMF. Here, important progress was achieved by the implementation of new assessments: While initial scales and questionaires, e.g. the Non-Motor Symptoms Scale (NMSS) and Questionaire (NMS-Quest) (Chaudhuri et al. 2006, 2007), the Non-Motor Fluctuation Assessment instrument (NoMoFA) (Kleiner-Fisman et al. 2011) as well as the MDS-UPDRS Part I (Non Motor Experiences of Daily Living) (Goetz et al. 2007; Martinez-Martin et al. 2013) quantified the severity and frequency of NMS, the MDS-Nonmotor Rating Scale (MDS-NMS) includes a standardized assessment of NMF in its NMF subscale (Chaudhuri et al. 2020). Besides specific scales, other assessments using patient diaries in combination with questionaires on motor fluctuations, e.g. the WOQ-19 (Stacy 2010) were applied. The newly developed MDS-NMS was recently used to determine the frequency of NMF using its NMF subscale in a sample of 402 PD patients with a mean disease duration of 8.2 years and in Hoehn & Yahr stages 1 to 4. 41% of these patients showed NMF, of whom 68,5% showed fluctuations in fatigue, 62,4% in anxiety, 40,6% in depression and 56,4% in thinking or cognitive abilities (Rodriguez‐Blazquez et al. 2021). A recent overview of scales used to assess NMF in PD patients was outlined by Boura et al. (Boura et al. 2025).

While the pathophysiology of motor fluctuations has been extensively investigated, the knowledge of underlying mechanisms of NMF is rather sparse. At least some NMF relate to impairments in the dopaminergic system, particularly for neuropsychiatric NMS (Ossig et al. 2016b), while others seem to originate from alterations in other neurotransmitter systems, such as serotonergic and noradrenergic activity (Classen et al. 2017). Anxiety and depression in PD correlate at least moderately with dopaminergic stimulation as fluctuations were predominantly associated with OFF states (Witjas et al. 2002; Ossig et al. 2016b), although OFF-related NMF do not precisely overlap temporally, with relatively longer durations of non-motor OFF periods (Ossig et al. 2017). The relevance of dopaminergic stimulation, and particularly of fluctuating pharmacokinetics was also highlighted in a study comparing oral levodopa versus jejunal application, where NMF were reduced in patients receiving continuous jejunal levodopa (Kulisevsky et al. 2020). Here, “episodic anxiety” not meeting criteria for an anxiety disorder was considered as unique to PD patients (Pontone et al. 2009). In the context of anxiety and depression as NMS, it is worth noting that ON-related euphoria is commonly associated with dopaminergic treatment (Delpont et al. 2017). Recent work on this PD specific fluctuating anxiety identified a subgroup of 31% of 200 patients with PD exhibiting anxiety exacerbation and more depressive symptoms, labeled as “anxious fluctuators” (Pontone et al. 2022). A comprehensive review confirmed the occurrence of these neuropsychiatric NMS in close association with motor OFF phases, while in patients without motor fluctuations the rate of neuropsychiatric NMF, particularly anxiety and mood, was very low (Velden et al. 2018).

Since a strong correlation of NMF with motor fluctuations is frequently present, optimization of dopaminergic treatment is an important strategy to reduce the burden of NMF. Therefore, most data from pharmacological interventions addressing NMF result from dopamine-enhancing therapies. Addition of the COMT-inhibitor opicapone or the MAO-B inhibitor safinamide, for example, reduced NMF (García et al. 2022; Masi et al. 2022). Further data on improving NMF result from studies with the dopamine agonists rotigotine (Chaudhuri et al. 2013) and pramipexole (Mueller et al. 2018). Besides specific physiotherapy (Ghielen et al. 2017), invasive therapies like continuous intrajejunal levodopa-carbidopa infusions as well as subthalamic nucleus deep brain stimulation seem to improve NMF in advanced stages of PD (Buongiorno et al. 2015; Ledda et al. 2024; Boura et al. 2025).

### Therapy of fluctuations: consideration of potential interactions

As the disease progresses, medication strategies must balance the complexities of deprescribing (Nguyen et al. 2024), addressing non-motor symptoms (Santos-García et al. 2020), and integrating adjunctive therapies. For example, while MAO inhibitors are often combined with selective serotonin inhibitors (SSRIs) to manage affective symptoms, next generation reversible MAO-B inhibitors at low SSRI doses present a minimal risk of serotonin syndrome (Aboukarr and Giudice 2018; Abbruzzese et al. 2020).

The use of anticholinergics in elderly or cognitively impaired patients poses additional concerns. Long-term studies link anticholinergics to heightened risks of mortality, falls, and dementia progression (Hong et al. 2019; Tan et al. 2020; Welk et al. 2021). However, much of the evidence is derived from non-selective agents, requiring careful interpretation. Concurrent administration of acetylcholinesterase inhibitors and anticholinergic agents, such as tricyclic antidepressants or atypical antipsychotics, is particularly problematic and should generally be avoided (Mantri et al. 2019).

### Addressing non-motor symptoms

Non-motor symptoms, such as sleep disturbances, provide another lens for therapeutic refinement. Commonly prescribed agents like Z-drugs and benzodiazepines carry significant risks, including dependence, paradoxical effects, and falls, particularly when combined with antidepressants (Martinez-Ramirez et al. 2016). Alternatives such as melatonin, optimized levodopa regimens, or pharmacokinetic adjustments using MAO-B (Bovenzi et al. 2024) or COMT inhibitors (Lees et al. 2016) may offer safer solutions. Non-pharmacological interventions like improved sleep hygiene (Dodet et al. 2024) are also crucial. Emerging treatments, such as orexin antagonists, show promise for improving sleep quality and enhancing overall well-being.

Over-medication, particularly with dopamine agonists, has been implicated in exacerbating sleep disorders, including excessive daytime sleepiness (Rosinvil et al. 2024). These findings highlight the importance of a balanced, individualized approach to treatment that adapts as the patient’s needs evolve.

### The evolving therapeutic landscape of Parkinson’s disease

The therapeutic landscape for Parkinson’s disease (PD) has advanced significantly, offering a diverse array of medications and combination strategies. This diversity is evident in both the frequency of daily dosages and the variety of active compounds available (Boucherie et al. 2021). Treatments are traditionally categorized into dopaminergic and non-dopaminergic agents, with dopaminergic therapies predominating. Tailoring therapy to individual needs involves considerations such as comorbidities, social factors, age, neuropsychiatric comorbidities, and prior adverse effects.

Innovative drug formulations have further diversified administration routes. Examples include transdermal systems like rotigotine (Nomoto et al. 2014), fast-dissolving and extended-release tablets (Hauser et al. 2011, 2023), and inhalable levodopa for rescue therapy (Farbman et al. 2020). Recently, subcutaneous delivery systems like foslevodopa (Rosebraugh et al. 2021) have expanded therapeutic options. In addition, levodopa pharmacokinetics can be modulated using inhibitors of key metabolic enzymes such as monoamine oxidase (MAO) and catechol-O-methyltransferase (COMT). These inhibitors vary in their side effect profiles and interaction risks, providing clinicians with nuanced options for fine-tuning treatment (Fabbri et al. 2022; Regensburger et al. 2023).

### On-demand therapies

On-demand therapies are an additional option when off episodes occur unpredictably, infrequently, or persist despite optimized dosing plans (Hauser et al. 2021; Pahwa et al. 2023). They may also be used for situations where patients anticipate the need for improved motor function during specific activities such as exercising or participating in social events. The current spectrum of on-demand treatments includes fast acting levodopa formulations, sublingual apomorphine patch, inhaled levodopa powder, and subcutaneous apomorphine. Availability of these options varies across countries. The choice of on-demand medication must be individualized, taking into account patient preferences, symptom profiles, timing and severity of off periods. The effectiveness of on-demand therapies depends on the patient’s ability to recognize an evolving off episode and to administer the medication promptly. This may require careful education (Olanow et al. 2021; Martinez-Nunez and LeWitt 2023; Isaacson et al. 2024).

### Low-risk strategies for optimized treatment

Non-pharmacological strategies represent essential adjuncts to pharmacological treatment, contributing to improved therapeutic efficacy and patient adherence. Interventions such as the management of chronic constipation (Carrasco et al. 2018), administration of probiotics (Tan et al. 2021), and dietary adjustments—including timing levodopa intake relative to protein-rich meals or adopting a low-protein diet (Rusch et al. 2023)—have demonstrated clinically meaningful benefits. While these approaches enhance therapeutic flexibility and personalization, they also introduce complexities related to regimen management and potential risks. Achieving an optimal balance between maximizing these benefits and mitigating associated challenges remains a key objective in the comprehensive management of PD.

### Device-aided therapies

While a tailored therapy for PD is desirable, it is not always feasible in practice. Challenges range from highly variable drug absorption throughout the day and external factors, such as temperature fluctuations, to seemingly simple issues like reduced compliance as treatment regimens grow more complex (Fleisher and Stern 2013). Device-aided therapies (DAT) offer a promising solution by simplifying regimens while enhancing efficacy (Antonini et al. 2023a, b). DAT might provide substantial symptom relief even allowing a monotherapy in some cases (Rožanković et al. 2024). These methods, which focus on continuous and targeted drug delivery, have demonstrated significant improvements in health-related quality of life. With careful consideration, DAT can be employed in various constellations in PD, providing an effective and manageable alternative for advanced disease stages. There were no substantial differences between different DAT alternatives regarding the absolute effects on motor fluctuations. However, the initiation of DAT requires careful evaluation of potential side effects, including neuropsychiatric complications from surgery or dopamine agonists, perioperative risks, and, importantly, the preferences and expectations of the patient which is paramount for therapy selection (Phokaewvarangkul et al. 2024). Table 1 provides a simplified overview of the most important effects as well as advantages and disadvantages of each DAT choice.

### Screening tools for device-aided therapies

Different algorithms have been proposed to identify those PD patients with fluctuations that benefit from DAT including.

- the expert consensus based “5-2-1” rule proposing criteria of “daily oral levodopa doses at least 5 times a day”, “at least 2 hours of the waking day with off symptoms” and “at least 1 hour of the day with troublesome dyskinesia” (Antonini et al. 2018),
- the Spanish CDEPA questionnaire comprising a matrix of symptom categories and their severity (Luquin et al. 2017),
- the “MANAGE-PD” tool (classifying into three categories) (Antonini et al. 2021),
- the “Navigate PD” consensus (compiling feedback from 103 experts) (Odin et al. 2015),
- the data driven D-DATS (Dutch Device Aided Therapy Screening) calculation tool based on levodopa equivalent daily dose and presence of troublesome dyskinesias and response to fluctuations (Moes et al. 2023a, b),
- the FLASQ-PD (Florida Surgical Questionnaire for Parkinson Disease) screening questionnaire to identify candidates for DBS (Okun et al. 2004),
- and the “Stimulus 1” and its updated version “Stimulus 2” electronic decision tools to identify DBS candidates (Moro et al. 2009; Wächter et al. 2011).

While it is evident that these screening algorithms do not account for medical contraindications as well as personal preferences of the patient and the neurologist, it is beneficial to familiarize with at least one of them. DAT are under-used in PD, and frequently, an earlier initiation would have had a beneficial effect on quality of life measures (Foltynie et al. 2024). Moreover, the implementation of these screening tools complements treatment guidelines when justifying a therapy choice for health insurance coverage (Smilowska et al. 2021, 2023).

**Table 1:**
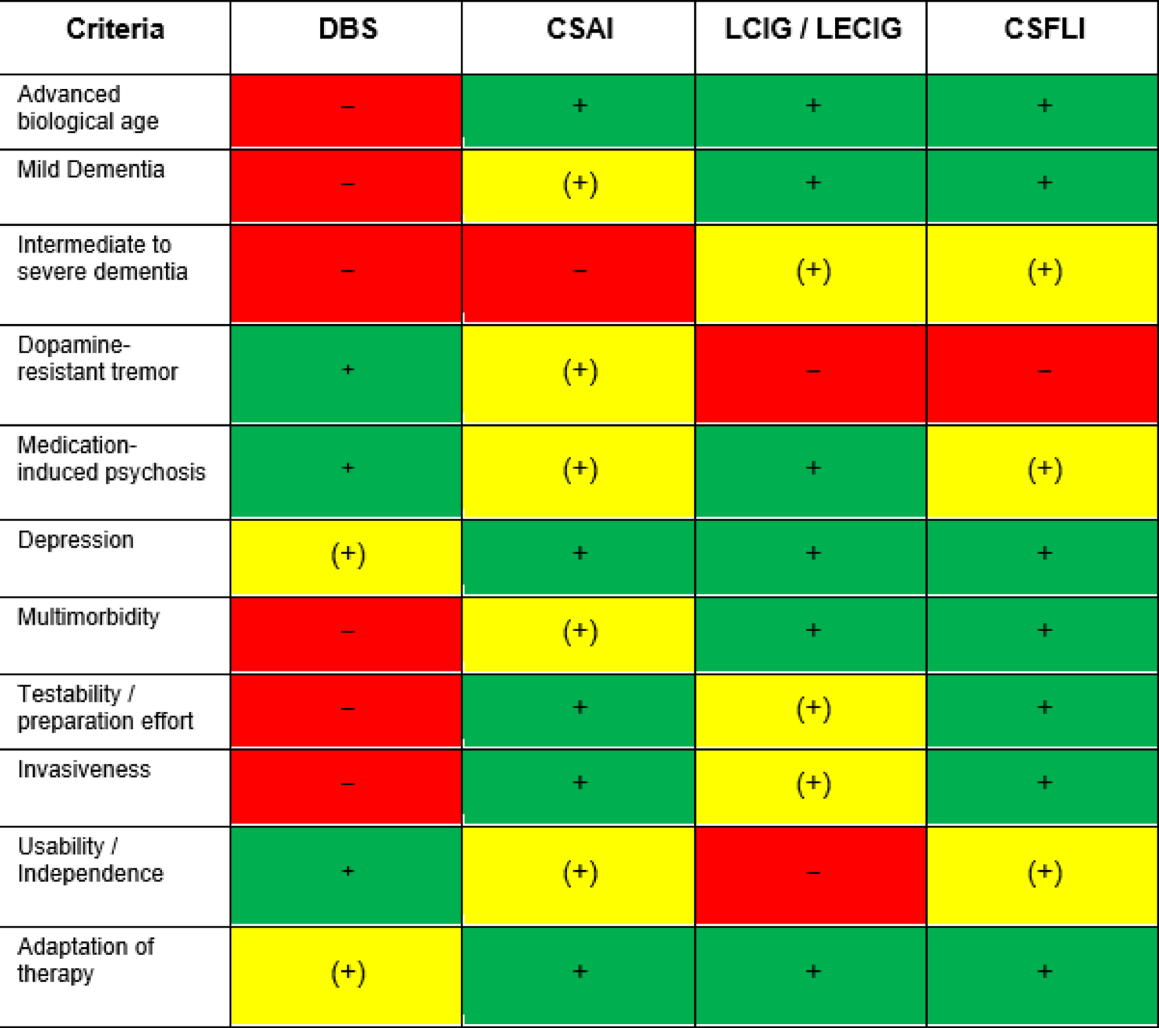
Characteristics of patient recommendations for device aided therapies (DAT) in PD with fluctuations, adapted from (Tönges et al. 2017; Deuschl et al. 2022; Demailly et al. 2024)

Note that comparisons are not based on direct head-to-head comparisons. *+ (green)* valid recommendation and / or positive evidence; *(+) (yellow)* limited recommendation and / or evidence; *– (red)* contraindication and / or negative evidence. *DBS* deep brain stimulation; *CSAI* continuous subcutaneous apomorphine infusion; *LCIG* levodopa carbidopa intestinal gel; *LECIG* levodopa entacapone carbidopa intestinal gel; *CSFLI* continuous subcutaneous foslevodopa/foscarbidopa infusion.

### Stem-cell based therapies

Two recent trials have offered glimmers of hope for the future of stem cell-based therapies in PD. These therapies could potentially target the same patient population as current device-aided therapies (Tabar et al. 2025; Sawamoto et al. 2025). While both studies are early-phase I/II trials with small patient numbers, focusing on safety and tolerability, they provide promising pilot data suggesting clinical efficacy that needs reproduction in larger cohorts and over longer follow-up periods. However, this approach requires stereotactic brain surgery, and it remains uncertain which patients would choose this invasive and irreversible procedure given the numerous available treatment options for managing PD fluctuations.

## Discussion and outlook

Although the development of a disease-modifying therapy remains an important unmet need in PD, significant advances have been made in symptomatic treatment options, particularly for motor symptoms. Typically, early motor symptoms respond well to dopaminergic treatment, but motor and non-motor fluctuations remain inevitable with ongoing neurodegeneration. A wide choice of different treatments is now available, and more will become so in the future. The lack of a standardized treatment regimen underscores the need for individualized therapeutic approaches. Comparative data between different dopaminergic therapies - particularly among device-aided treatments including DBS - are limited and it is unclear whether head-to-head studies with these therapies will ever be performed. This stresses the necessity to develop non-invasive dopaminergic treatment options combining rapid and extended symptom relief. Furthermore, much better management is needed for non-motor symptoms, where therapeutic options are still limited. Last but not least, even advanced medications and DAT have to compete with the continuous progression of the disease and in rare cases, combinations of DAT are required. First evidence suggests that the combination of DAT may be as efficient as the initial placement of a DAT (Pürner et al. 2023), but larger and prospective studies are needed to provide better evidence here.
